# Thermal and aqueous stability improvement of graphene oxide enhanced diphenylalanine nanocomposites

**DOI:** 10.1080/14686996.2016.1277504

**Published:** 2017-02-23

**Authors:** Kate Ryan, Sabine M. Neumayer, Harsha Vardhan R. Maraka, Nicolae-Viorel Buchete, Andrei L. Kholkin, James H. Rice, Brian J. Rodriguez

**Affiliations:** ^a^ School of Physics, University College Dublin, Dublin, Ireland; ^b^ Conway Institute of Biomolecular and Biomedical Research, University College Dublin, Dublin, Ireland; ^c^ Institute for Discovery, University College Dublin, Dublin, Ireland; ^d^ Department of Physics, CICECO-Aveiro Institute of Materials, Aveiro, Portugal; ^e^ Institute of Natural Sciences, Ural Federal University, Ekaterinburg, Russia

**Keywords:** Diphenylalanine, nanocomposites, peptide nanotubes, graphene oxide, aqueous stability, 30 Bio-inspired and biomedical materials, 100 Materials, 101 Self-assembly / Self-organized materials, 103 Composites, 104 Carbon and related materials

## Abstract

Nanocomposites of diphenylalanine (FF) and carbon based materials provide an opportunity to overcome drawbacks associated with using FF micro- and nanostructures in nanobiotechnology applications, in particular their poor structural stability in liquid solutions. In this study, FF/graphene oxide (GO) composites were found to self-assemble into layered micro- and nanostructures, which exhibited improved thermal and aqueous stability. Dependent on the FF/GO ratio, the solubility of these structures was reduced to 35.65% after 30 min as compared to 92.4% for pure FF samples. Such functional nanocomposites may extend the use of FF structures to e.g. biosensing, electrochemical, electromechanical or electronic applications.

## Graphical abstract

**Figure:**
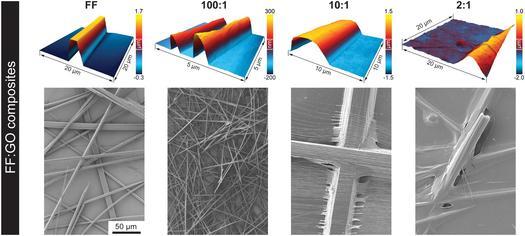

## 1. Introduction

The combination of materials into nanocomposites provides unique design possibilities, potentially leading to, for example, high performance biomimetic materials comprising biopolymers and nanomaterials at the nanometer scale [1, 2]. Whereas carbon based nanomaterials such as carbon nanotubes (CNTs) can modify mechanical, thermal, and electrical properties [3–5] of a nanocomposite [6, 7], the biocompatibility and biodegradability of biopolymers can unlock biomedical applications for e.g. tissue engineering [8].

Due to their ability to hierarchically self-assemble from functional molecular units into micro- and nanostructures, peptides are ideal candidates for nanoarchitectonics-based material design [9, 10]. Tuning the preparation conditions allows tailored self-organized formations such as fibers, rods, tubes, ribbons, and crystals to be obtained [11–13]. In particular, bioinspired peptide nanostructures such as diphenylalanine (FF) micro- and nanotubes have been actively researched for nanotechnology and biomedical applications [14–17]. FF nanotubes are known for their high mechanical strength, piezoelectric properties, and excellent functionalization capabilities, making them attractive materials for biosensing, tissue engineering, and energy harvesting applications [14–16, 18]. The instability of FF nanotubes in solution is a major limitation to realizing FF nanotube-based biosensors or drug delivery systems [19, 20] and routes to overcome this barrier that maintain their properties are required.

Carbon based nanomaterials such as CNTs, graphene, and graphene oxide (GO) have been widely used in nanocomposites because of their nanoscale dimensions and their respective mechanical, electrical, and chemical functionalization properties [3–6, 21], and in some cases have been combined with peptides. Montenegro et al. [22] reported improved stability in aqueous solution and electrical conductivity of a dual composite of single walled CNTs and self-assembling cyclic peptide nanotubes and Yuan et al. [23] developed a nanocomposite of multi-walled CNTs covered by FF peptides, which was subsequently used to coat an electrode for high-sensitivity ethanol biosensing. Additionally, Chen et al*.* [24] reported the functionalization of CNTs by aromatic molecules as a template for the immobilization of biological molecules. Furthermore, layered composite structures of electrostatically interacting amyloid fibers and graphene have been shown to have a tunable shape memory effect attributed to the increased elastic strength provided by the reinforcing amyloid fibers [25]. With regards to GO, it has previously been combined with piezoelectric materials ZnO and poly(vinylidene fluoride) to produce piezoelectric composite materials with increased functionality [26, 27] and has been used in applications requiring a high degree of biocompatibility, such as intracellular probing of living cells [28], DNA biosensing [29], glucose biosensing [30], and drug delivery [31]. Notably, Han et al*.* [32] fabricated reduced graphene oxide-coated peptide nanowires via self-assembly. Given the abilities of GO to form piezoelectric composites and to function in biological applications, it is a promising material for carbon-based/FF nanotube composites.

In this work, layered FF/GO nanocomposite materials were investigated to establish whether the main limitation associated with FF micro- and nanotubes, i.e. their relatively poor stability in solution, could be overcome. Additionally, the morphological, structural, and thermal modifications resulting from the addition of GO were characterized using scanning electron microscopy (SEM), atomic force microscopy (AFM), Raman spectroscopy, and thermogravimetric analysis (TGA). In this study, the possibility of improving the aqueous stability of FF micro- and nanotubes through addition of GO was examined using *in situ* optical microscopy of FF and composite FF/GO structures when immersed in water.

## 2. Materials and methods

### 2.1. Sample preparation

Solutions of GO with platelet thickness 0.7–1.2 nm (Cheap Tubes, USA) and DI water with concentrations of 2 mg ml^−1^, 0.2 mg ml^−1^, and 0.02 mg ml^−1^ were prepared by serial dilution. A stock FF solution of 100 mg ml^−1^ FF (Bachem, UK) in hexafluoroisopropanol (Sigma Aldrich, Ireland) was then diluted to 2 mg ml^−1^ with each of the GO/DI water solutions to give solutions containing GO:FF concentrations of 1:100, 1:10, 1:2, and 1:1 (Table 1). Solutions consisting solely of FF (2 mg ml^−1^) and GO (2 mg ml^−1^) were also prepared. For Raman, TGA, and stability investigations, samples were prepared by depositing 30 μl of each solution onto glass coverslips and drying under ambient conditions (21°C, humidity ~ 30%) for 24 h. For AFM measurements, 10 μl was deposited onto glass cover slips prior to drying under the same conditions.

**Table 1.** Ratios and weights of solutions used for sample preparation.

Sample name	Ratio		Weight FF [mg]	Weight GO [mg]
	FF	GO		
100:1	100	1	4	0.04
10:1	10	1	4	0.4
2:1	2	1	4	2
FF	100	0	4	0
GO	0	100	0	4

### 2.2. Sample characterization

SEM: Samples were imaged using a Hitachi S-4300 (Japan) field emission scanning electron microscope.

AFM: AFM measurements were performed in contact mode using an atomic force microscope (MFP-3D Asylum Research, USA) and cantilevers (MikroMasch, HQ:DPE-XSC11 lever C, Bulgaria) with a nominal spring constant of 7 N m^−1^.

Raman spectroscopy: Raman spectra were collected at room temperature using a Raman micro-spectrometer based on an inverted microscope (Olympus, Japan) as outlined in previous reports [33–37]. A green Ar-ion laser light source at a wavelength of 532 nm was used as the excitation wavelength. Signals were collected using backscattering geometry onto an electron multiplying charge coupled device via a monochromator. The resolution of the spectrometer was 0.5 cm^−1^. The diameter of the beam spot was ∼15 μm and the power was less than 3 mW in order to avoid sample degeneration caused by laser heating. The Raman spectrum acquisition time set to 100 s using Andor SOLIS software (http://www.andor.com/scientific-software).

TGA: TGA was carried out using a TA Instruments Q500 (USA) series at a ramp rate of 10°C from 25°C up to 800°C under an air flow of 100 ml min^−1^.

Aqueous stability study: Each sample was solvated with DI water to study aqueous stability. The samples were imaged at the same location at time intervals of 60 s over 35 min using an optical microscope (Nikon, Japan). The microscope illumination was switched off between each image acquisition to minimize thermal effects.

The optical images were analyzed using ImageJ image processing and analysis software [38]. The images were converted to binary images using an automatic threshold level based on iterative intermeans. This algorithm divides the image into object and background by taking an initial threshold and computes the averages of the pixels at or below the threshold. Subsequently, the threshold is incremented and the process is repeated until the threshold is larger than the average of all pixels [39]. The histogram of the distribution of gray values in the resulting binary image, indicates the number of white and black points, where white points correspond to the amount of visible substrate surface and black to the area covered by tubes. By assessing the coverage prior to immersing in water it was possible to estimate the percentage loss in coverage, relating to the amount of tubes which are dissolved.

## 3. Results and discussion

### 3.1. Morphology

**Figure 1.**
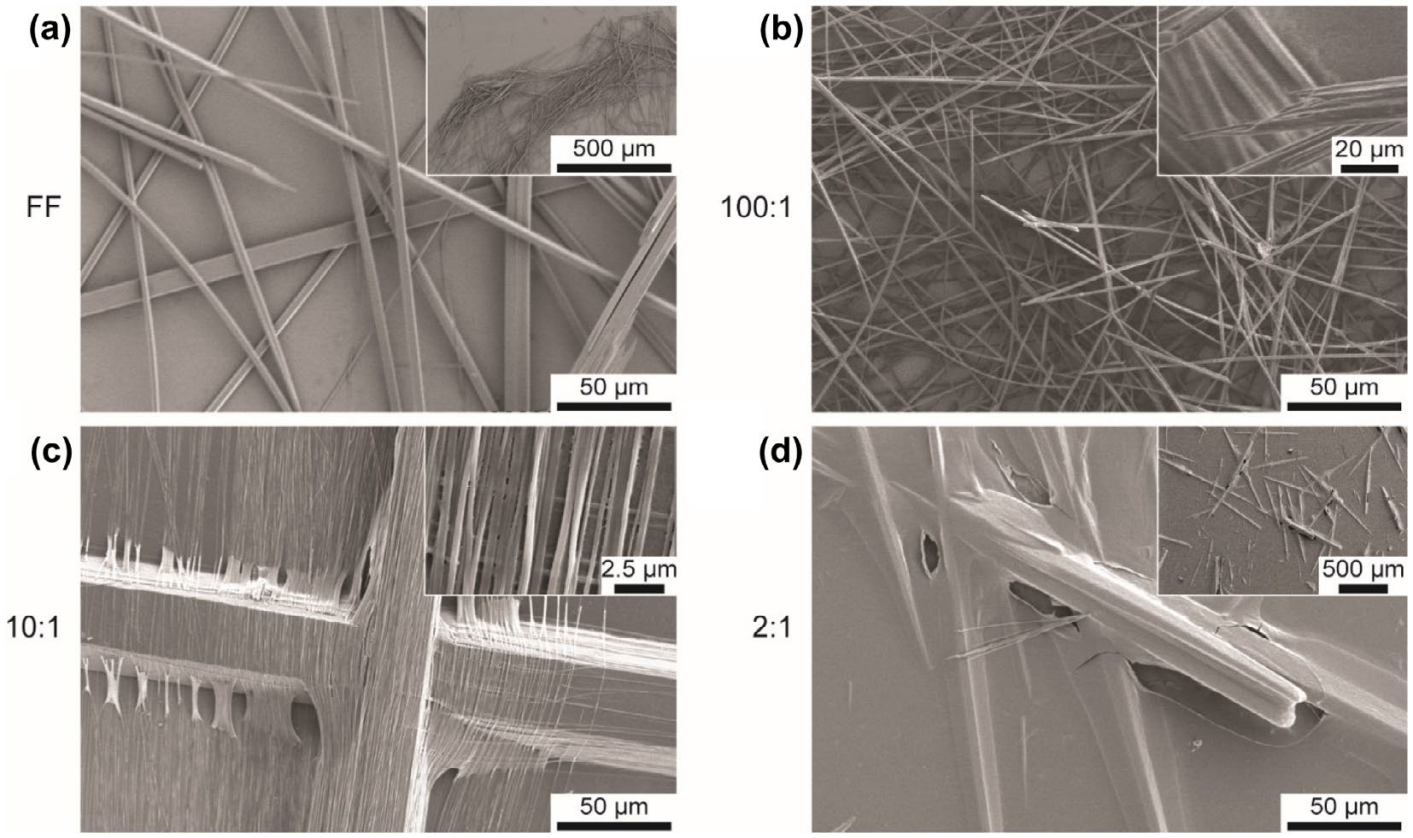
SEM images of samples (a) FF, (b) 100:1, (c) 10:1 and (d) 2:1.

**Figure 2.**
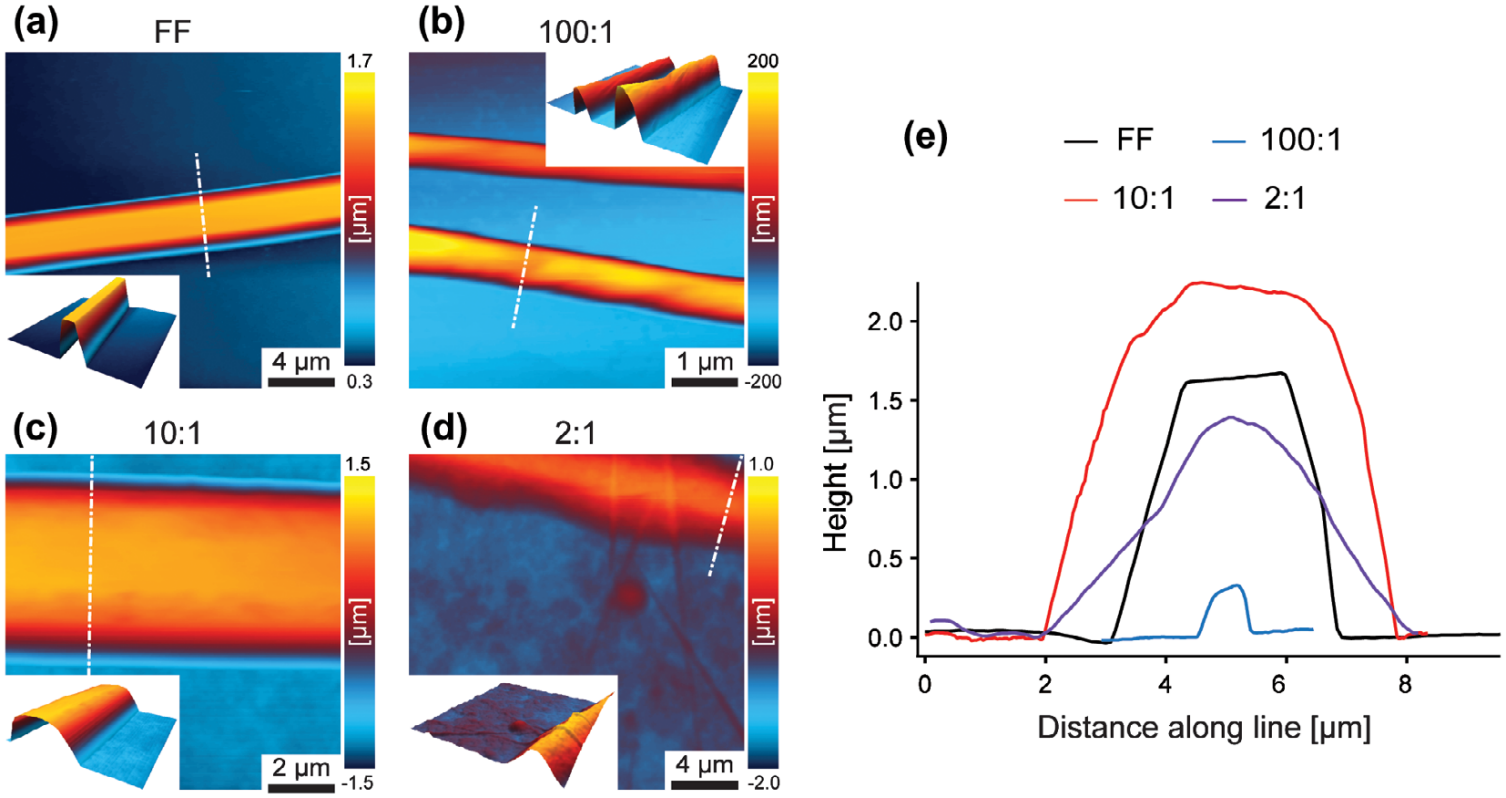
AFM height images of samples (a) FF, (b) 100:1, (c) 10:1 and (d) 2:1 with three-dimensional illustrations as insets. (e) Cross section profiles of samples FF, 100:1, 10:1 and 2:1 extracted as indicated in (a–d) by white dashed lines (x- and y- offsets applied).

To investigate the formation of FF:GO nanocomposites and to assess their size and shapes, the prepared samples have been imaged using SEM (Figure 1) and AFM (Figure 2). FF in the absence of GO forms a typical network of micro- and nanotubes of varying diameter (2–15 μm) and length (Figure 1(a)). Addition of GO to the sample solution appears to impact the morphology of structures; however, self-assembly into tubes persists. Tubes on sample 100:1 appear thinner than on FF with diameters between 1–5 μm (Figure 1(b)). As depicted in Figure 1(c), increasing the amount of GO to 10:1 leads to peptide bundles (diameter ~50 μm) as well as smaller individual tubes (diameter ~ tens of nm). On sample 2:1, a GO layer is covering the peptide tubes and the substrate (Figure 1(d)). The diameters of the tubes vary significantly, ranging from 500 nm to 20 μm.

In order to further investigate the morphology of single tubes and explore the role of GO, AFM was employed. Apart from the size of the tubes, also the shape changes with increasing GO content (Figure 2). As visible in AFM height images (Figure 2(a)–(d)) and corresponding cross section profiles (Figure 2(d)), the FF tube exhibits a flat surface and pronounced edges, consistent with other reports [18]; however, with increasing GO content, the shape changes and becomes increasingly round. Furthermore, GO aggregates are observed on the substrate of sample 10:1 and, more pronounced, on sample 2:1, whereas the substrates of the FF and 100:1 sample do not exhibit such deposition. These observations indicate that GO is predominately located on the tube surface, as reported elsewhere [32], with the degree of coating depending on the GO content. As the basal plane of GO is reported to be more hydrophobic than the hydrophilic edges [40], noncovalent assembly of peptides with hydrophobic and aromatic residues are expected [32, 41–44]. Han et al. [32] reported that peptide nanotube core reduced graphene oxide shell nanowire assembly occurred only when the peptide nanotubes and reduced graphene oxide sheets had opposite charge, suggesting a dominant role of electrostatics in the formation of those structures.

### 3.2. Molecular interactions

**Figure 3.**
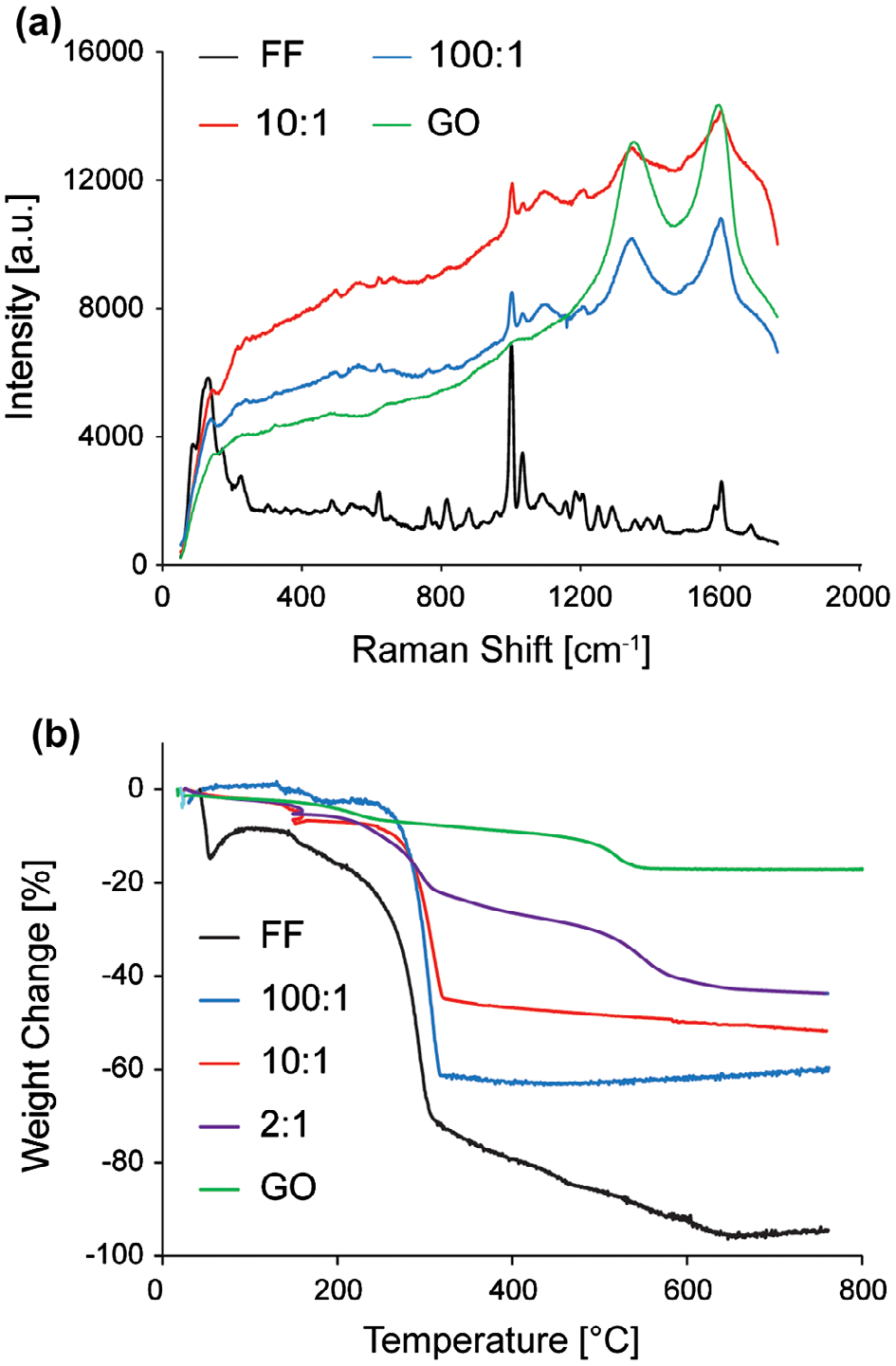
(a) Raman spectra of FF, GO and composite samples 100:1 and 10:1. (b) TGA thermogram of FF, GO, and samples 100:1, 10:1 and 2:1.

FF/GO nanocomposites have been characterized by Raman spectroscopy to establish whether the molecular structure of the FF tubes has been altered with the addition of GO. Figure 3(a) shows the Raman spectra for native phase FF tubes, GO only and FF/GO nanocomposites.

Graphitic materials exhibit a highly dispersive band in the 1200–1400 cm^−1^ region, known as the disorder-induced D band and the graphitic mode (G) band at higher frequencies (~1590 cm^−1^), due to the stretching of sp^2^ atoms in the rings and chains [45, 46]. The sample containing only GO exhibits two distinct bands at 1350 and 1597 cm^−1^, in agreement with the literature. The major Raman peaks associated with FF tubes are also exhibited in the FF only spectrum. The bands at 1002 and 1033 cm^−1^, 1184 and 1205 cm^−1^, and 1583 and 1603 cm^−1^ correspond to aromatic vibrations. Amide III (CN) bands are at 1249 and 1287 cm^−1^ and the amide I band (CO) appears at 1687 cm^−1^ [47].

The spectrum captured on composite samples exhibits FF and GO modes. For 100:1 tubes, the aromatic 1002 and 1033 cm^−1^ bands are reduced compared to those detected for FF and the amide I band at 1688 cm^−1^ associated with CO stretching [48] is absent. For 10:1 there are further notable changes to the Raman spectra. The aromatic band at 1205 cm^−1^ associated with CH in-plane bending of the aromatic ring [49] is also absent. The amide III bands at 1290 cm^−1^ (relating to CN stretch and NH in-plane bending) are not visible in the spectra for either nanocomposite sample. This could be a result of the relatively higher intensity of the disorder-induced D band of GO, which is in the same frequency range [45, 49].

The presence of the respective characteristic Raman bands indicate that the corresponding molecules are present in the volume of material excited by the laser. The disruption to the CH band at 1205 cm^−1^ indicates the possible disruption to the FF structure by the presence of GO, by interaction with the aromatic rings. FF molecules are most likely to interact by hydrogen bonding at the NH and not at the CO sites, determined by the frequencies of the amide III and amide I bands [47]. The shifting of these bands could indicate the disruption of the structure of the FF nanotube. Furthermore, the shift of the amide bands with addition of GO becomes more pronounced with increasing concentration. Previously, it was demonstrated that wrapping of collagen structures with CNTs shifts the vibrational characteristics of the sample, which causes shifts in the spectra [50–52]. Therefore, these results indicate molecular interactions between the FF and GO components; however, location and level of the interaction between the FF and GO remain to be determined.

During Raman measurements, local light induced bleaching behavior was observed for sample 2:1, a phenomenon associated with sample damage which is not observed for GO or FF in their native states as both are thermally stable structures. Therefore, combining GO and FF appears to lead to significant changes to the thermal properties of composite FF/GO structures.

### 3.3. Thermal stability

The alteration of thermal properties in FF/GO composites as observed during Raman spectroscopy was further investigated using TGA. The thermogram for native FF tubes (Figure 3(b)) shows an initial weight decrease (~11.9%) over a temperature range of 10.0–49.4°C, which is likely to result from initial evaporation of water molecules [53]. A major decrease in weight can be observed at 169.4°C in agreement with an initial release of phenylalanine from FF tubes reported in the literature [54]. There is a rapid loss in weight between 223.3°C and 310.5°C, from –11.9% to –70.7%, and the thermal decomposition appears to be complete with 95.8% loss at ~ 652.4°C.

The thermal behavior changes upon addition of GO to the FF structure. Sample 100:1 exhibits a slower degradation, with an onset at 185.0°C and two step-wise weight loss events commonly observed for nanocomposite materials [55]. The major weight loss event is comparable to the native FF tubes, occurring at 235.4°C, and the final weight loss was observed at –59.2%. The sample prepared with a ratio of 10:1 displays a similar degradation profile with less overall weight loss. Thermal decomposition is complete at 255°C, with a final loss 52% of at 800°C. Sample 2:1 adopts a similar profile to the native GO thermogram and displays a gradual loss of moisture between 10.0 and 178.9°C. Thermal degradation is complete at 597°C with an overall weight loss of –40.9%. The GO thermogram displays the characteristic behavior for GO, exhibiting loss of a large amount of H_2_O molecules and oxygen-functional groups from the basal plane, with an overall weight loss of 17% up to 800°C [56]. In order to compare the thermogram for different samples, the varying initial water content has to be taken into account, which is likely to be affected by the addition of GO. Nevertheless, the results indicate that increasing the amount of GO leads to less weight loss overall, suggesting a higher stability at higher temperatures.

### 3.4. Aqueous stability

**Figure 4.**
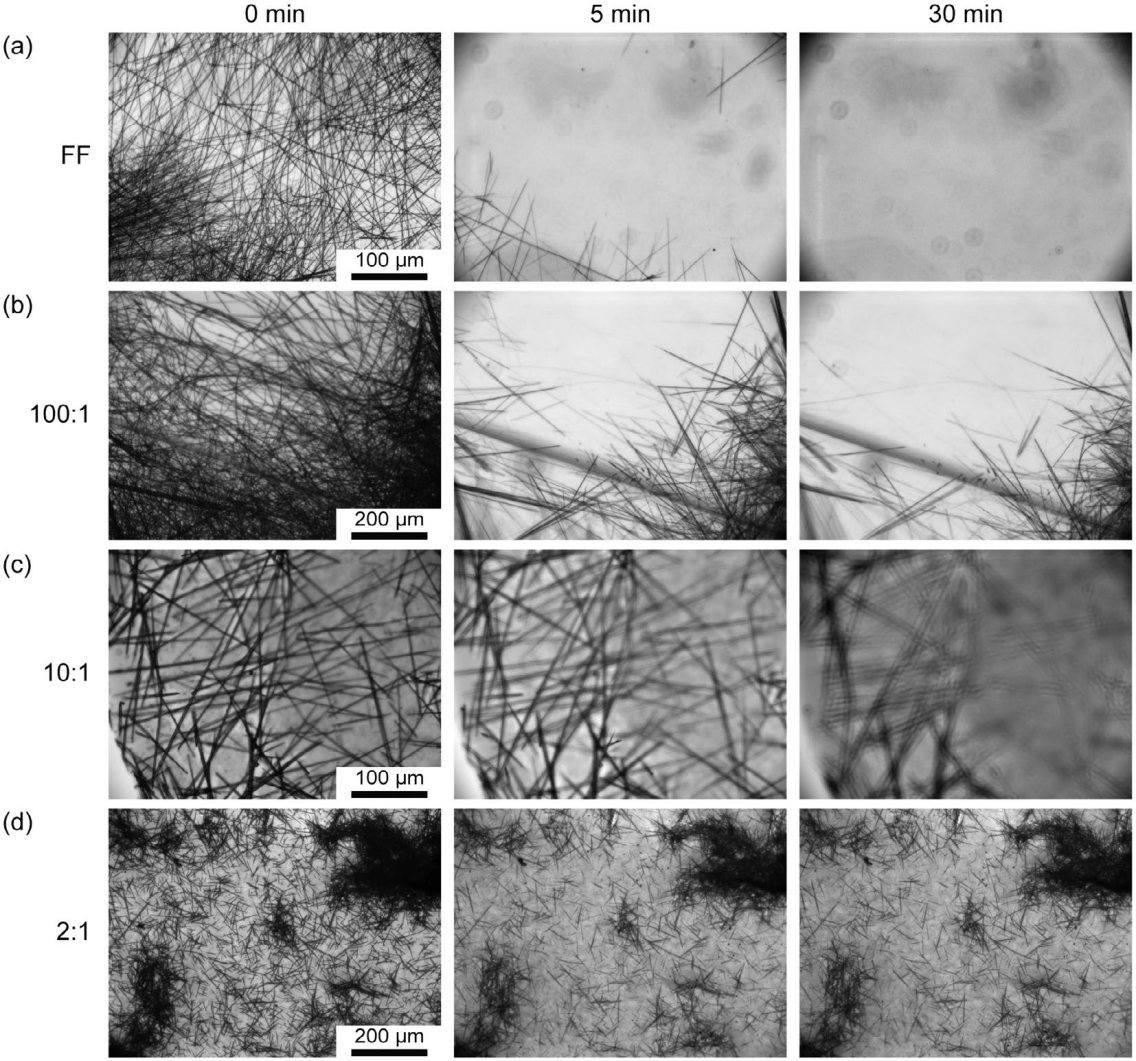
Optical images showing aqueous stability of FF/GO nanocomposites after 0 min, 5 min, and 30 min for (a) FF, (b) 100:1, (c) 10:1, and (d) 2:1 samples.

The increased thermal stability of FF/GO composites prompted the question whether increased stability is also observed in water. As depicted in Figure 4(a) and Table 2, after only 5 min 90.6 ± 0.1% of FF tubes were already dissolved which increased to 92.44 ± 5.9% after 30 min. Sample 100:1 exhibits higher aqueous stability (Figure 4(b)) with only 81.7 ± 4.1% and 63.9 ± 7.5% dissolved after 5 and 30 min, respectively. From Figure 4(c), a significant decrease in solubility of sample 10:1 can be observed. The high percentage of dissolved tubes after 30 min in Table 2 results from a difficulty in maintaining the focus throughout the time lapse which leads to an overestimation of the % loss in coverage. However, most tubular structures present in the 5 min image can also be identified after 30 min. Increasing the GO content to a ratio of 2:1 further inhibits dissolvability (Figure 3(d)) with only 33.3 ± 10.9% of the sample dissolved after 5 min. This percentage stays approximately constant and is 35.6 ± 10.4% after 30 min.

**Table 2.** Percentage loss in coverage when samples were placed in DI water. Data shown for 0, 5, and 30 min.

Sample	% dissolved – water stability		
	0 min	5 min	30 min
FF	0%	90.6 ± 0.1%	92.4 ± 6.0%
100:1	0%	63.9 ± 7.5%	81.7 ± 4.1%
10:1	0%	30.1 ± 9.9%	50.3 ± 5.0%
2:1	0%	33.3 ± 10.9%	35.6 ± 10.4%

These findings suggest that the poor aqueous stability of FF tubes that is limiting especially for biomedical applications, may be overcome to a significant extent upon addition of GO. The increased stability likely stems from the capping layer formed by GO that reduces interaction between water and peptide molecules.

## 4. Conclusions

GO and FF were combined to form layered structures exhibiting significantly reduced aqueous solubility and altered thermal properties as compared to pure FF tubes. These composite structures exhibited improved thermal stability resulting from a slower onset of degradation and less overall % weight loss. Furthermore, an improved aqueous stability with increasing GO content was observed with only 35.65% of 2:1 FF:GO structures being dissolved after 30 min as compared to 92.4% of native FF tubes. GO enhanced FF nanostructures could therefore provide a path for peptide-based structures with broad nanotechnology applications such as energy storage and photovoltaics [57–60].

## Disclosure statement

No potential conflict of interest was reported by the authors.

## Funding

The work was supported by the European Commission within FP7 Marie Curie Initial Training Network ‘Nanomotion’ [grant agreement no. 290158]. Part of this work was supported by Science Foundation Ireland [14/US/I3113 and SFI07/IN1/B931]. The work was partially supported through the DGPP which was funded under the Programme for Research in Third Level Institutions (PRTLI) Cycle 5 and co-funded by the European Regional Development Fund. The work was also supported in part by the Russian Foundation for Fundamental Research [16-29-14050] and by the CICECO-Aveiro Institute of Materials [POCI-01-0145-FEDER-007679; FCT Ref. UID/CTM/50011/2013], financed by national funds through the FCT/MEC and co-financed by FEDER under the PT2020 Partnership Agreement.
